# Nomogram predicting the efficacy of transurethral surgery in benign prostatic hyperplasia patients

**DOI:** 10.1007/s40520-024-02708-8

**Published:** 2024-03-14

**Authors:** Jing Zhou, Zhu-Feng Peng, Lu-Chen Yang, Sheng-Zhuo Liu, Pan Song, Zheng-Huan Liu, Lin-Chun Wang, Jun-Hao Chen, Kai Ma, Yun-Fei Yu, Liang-Ren Liu, Qiang Dong

**Affiliations:** 1 Department of Urology, West China Hospital, Sichuan University, No. 37, Guoxue Alley, Chengdu, 610041 Sichuan People’s Republic of China; 2 Institute of Urology, West China Hospital, Sichuan University, Chengdu, 610041 China; 3 West China School of Medicine, Sichuan University, Chengdu, China

**Keywords:** Benign prostatic hyperplasia, Transurethral surgery, Prediction model, Nomogram

## Abstract

**Purpose:**

This study aimed to develop and validate a nomogram for predicting the efficacy of transurethral surgery in benign prostatic hyperplasia (BPH) patients.

**Methods:**

Patients with BPH who underwent transurethral surgery in the West China Hospital and West China Shang Jin Hospital were enrolled. Patients were retrospectively involved as the training group and were prospectively recruited as the validation group for the nomogram. Logistic regression analysis was utilized to generate nomogram for predicting the efficacy of transurethral surgery. The discrimination of the nomogram was assessed using the area under the receiver operating characteristic curve (AUC) and calibration plots were applied to evaluate the calibration of the nomogram.

**Results:**

A total of 426 patients with BPH who underwent transurethral surgery were included in the study, and they were further divided into a training group (*n* = 245) and a validation group (*n* = 181). Age (OR 1.07, 95% CI 1.02–1.15, *P* < 0.01), the compliance of the bladder (OR 2.37, 95% CI 1.20–4.67, *P* < 0.01), the function of the detrusor (OR 5.92, 95% CI 2.10–16.6, *P* < 0.01), and the bladder outlet obstruction (OR 2.21, 95% CI 1.07–4.54, *P* < 0.01) were incorporated in the nomogram. The AUC of the nomogram was 0.825 in the training group, and 0.785 in the validation group, respectively.

**Conclusion:**

The nomogram we developed included age, the compliance of the bladder, the function of the detrusor, and the severity of bladder outlet obstruction. The discrimination and calibration of the nomogram were confirmed by internal and external validation.

**Supplementary Information:**

The online version contains supplementary material available at 10.1007/s40520-024-02708-8.

## Introduction

Benign prostatic hyperplasia (BPH) is one of the most common urologic diseases, and it is also one of the main disorders causing lower urinary tract symptoms in aging males [1]. With the increasing life expectancy of male people, the number of elderly patients with BPH is also increasing. To date, transurethral surgery is one of the most effective ways for the treatment of BPH [2]. Whether it is possible to improve their urination status and quality of life through transurethral surgery is one of the most concerning issues for patients and clinicians before surgery. However, the prediction of the recovery of postoperative micturition function mainly depends on the experience of the surgeon. Currently, there are increasing studies focusing on preoperatively predicting the efficacy of BPH surgery; nevertheless, most studies have only applied one predictor for the prediction of the recovery of voiding function in patients with BPH after transurethral surgery [3, 4]. Additionally, previous studies revealed that the efficacy of transurethral surgery is affected by multiple factors [5–7]. It is obvious that the degree of influence of each predictor on urination recovery after BPH cannot be determined; besides, there may be a variety of unreported potential factors, thus predicting the patient's surgical outcome via a single predictor may not be ideal. Therefore, identifying predictors with a strong predictive ability and developing a risk model to predict the efficacy of transurethral surgery is of vital importance.

Nomogram is a graphical predictive method that combines different variables to generate a continuous scoring system and calculates the risk probability of a clinical event for each patient [8]. It meets clinical requirements for an integrated model, playing a significant part in the personalized treatment for patients and the prognosis prediction for clinicians. The nomogram has been successfully used in predicting lymph node metastasis and mortality of prostate cancer or bladder cancer in recent years [9, 10]. To date, a nomogram with enough statistical power to predict the recovery of voiding function in patients after surgery has yet to be designed.

In this study, we aimed to identify the predictors for the efficacy of transurethral surgery and establish a novel nomogram with internal and external validation for predicting the efficacy of transurethral surgery in BPH patients using data from the West China Hospital and West China Shang Jin Hospital.

## Methods

### Study design and participants

Patients included in the training group and the validation group underwent transurethral surgery from January 2019 to June 2022 at West China Hospital, Sichuan University and Shang Jin Hospital. Patients in the training group and validation group underwent greenlight photoselective vaporization of the prostate (PVP) and transurethral resection of the prostate (TURP). Patients who met the following inclusion criteria were recruited in this study: (1) diagnosis of lower urinary tract symptoms due to BPH causing BOO which is refractory to conservative therapy; (2) international prostate symptom scores > 12. The exclusion criteria were as follows: (1) suspect of prostatic or other cancer; (2) diagnosed with a urethral stricture; (3) diagnosis of neurogenic bladder or other neurologic disorder; and (4) history of bladder outlet obstruction surgery.

### Data collection and variables recorded

Baseline characteristics included age, body mass index (BMI), lower urinary tract symptoms (LUTS) duration, International Prostate Symptom Score (IPSS), quality of life (QoL) score, history of urinary retention, and comorbidities including hypertension and diabetes. Preoperative characteristics included prostate volume (PV), post-void residual (PVR), and total prostate-specific antigen (tPSA), and urodynamic results included maximum flow rate (Qmax), bladder compliance, the function of detrusor, and the severity of bladder outlet obstruction (BOO). Urodynamic studies were carried out in the sitting position with a Laborie Triton equipment using the air‐charged catheters (Laborie &Co., version: Aquarius TT Triton) following GUP guidelines [11, 12]. We defined detrusor underactivity as a formulated bladder contractility index (BCI = PdetQmax + 5 Qmax) < 100. As for bladder compliance, the compliance was calculated based on the methods described previously [12], and the bladder with compliance < 15 ml/cm H_2_O was considered as non-compliant [13]. With regard to the BOO, the bladder outlet obstruction index (BOOI) was used (BOOI = PdetQmax − 2Qmax) and BOOI > 40 was considered as obvious BOO [14]. All patients were in a routine visit follow-up at 6 months after discharge, and patients with postoperative IPSSs more than 10 or Qol scores more than 3 and postoperative IPSS decrease < 10 were determined as incomplete recovery [4, 15, 16].

### Statistical analysis

Categorical variables were expressed as numbers (percentage) and continuous variables were expressed as medians with ranges or means with standard deviations. Differences in baseline characteristics between groups for continuous variables were assessed using the variance analysis. Multivariate logistic regression analysis with the forward selection method was applied to determine the major predictors in the nomogram. Variables included in the multivariate logistic regression were those with a *P* value of < 0.05 in the univariate analysis. Variance Inflation Factor (VIF) was utilized for collinearity diagnosis of the covariates in the model, with tolerance < 0.1 and VIF > 4 considered indicative of multicollinearity.

In the nomogram, points were assigned by drawing a vertical line from the corresponding values of each predictor to the “Points” axis. The total point is the cumulative sum of the points assigned to each predictor. The probability of incomplete postoperative recovery is obtained by drawing a vertical line from the “Total points” axis to the “Risk” axis. The discriminative performance of the nomogram was measured by calculating the area under curve (AUC) of the receiver operating characteristic curve (ROC). Calibration was tested using a calibration plot, which described the fitting degree between actual and nomogram-predicted incomplete recovery. Then the nomogram was constructed by using the RMS package in the R (r4.1.3) software to visually score individual risk probabilities.

## Results

**Fig. 1:**
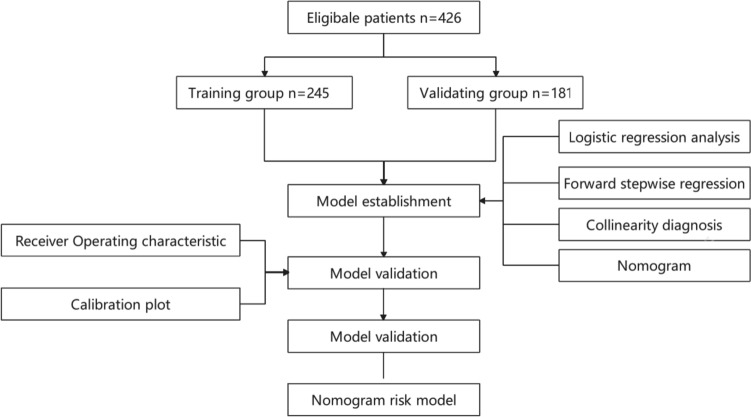
Flow chart of the study design and analysis

The flow chart of patient inclusion is presented in Fig. 1. From July 2019 to June 2022, a total of 245 patients treated with transurethral surgery were included in the training group. And a total of 181 patients were included in the validation group. Detailed characteristics of the two groups are shown in Table 1. The number of patients had incomplete recovery in the training group and validation group was 65 (27%) and 52 (28%), respectively.

**Table 1:** Clinical characteristics of patients in training and validation groups

	Training group (*n* = 245)	Validation group (*n* = 181)	*P* value
Age (years)	68.1 ± 8.9	69.1 ± 7.8	0.23
BMI (kg m^2^)	23.6 ± 3.0	24.1 ± 3.0	0.07
Duration (months)	36.0 (12.0, 72.0)	36.0 (12.0, 63.0)	0.86
IPSS	24.8 ± 3.3	24.7 ± 3.3	0.70
Qol	5.2 ± 0.6	5.1 ± 0.9	0.13
tPSA ng/ml	4.1 (2.3, 7.3)	4.2 (2.4, 6.7)	0.43
PV (ml)	64.7 (60.6–65.9)	63.1 (60.3–65.8)	0.92
PVR (ml)	85.6 (75.1–96.9)	75.8 (63.4–88.3)	0.23
Qmax (ml/s)	8.29 (7.05–9.52)	7.99 (7.37–8.61)	0.70
MCC (ml)	209.4 ± 47.9	199.2 ± 52.4	0.37
Surgery type			
PVP	157	122	0.48
TURP	88	59	
Hypertension			0.76
Yes	34	27	
No	211	154	
Diabetes			0.33
Yes	32	28	
No	213	153	
Bladder compliance			
Decompensation	73	60	0.46
Normal	172	121	
Detrusor function			
Decompensation	153	114	0.91
Normal	92	67	
Bladder outlet obstruction			
Obvious	113	79	0.61
Unclear	132	102	

*BMI* Body Mass Index, *PV* Prostate Volume, *PVR* Post-void Residual, *MCC* Maximum Cystometric Capacity, *Qmax* maximum flow rate, *tPSA* total prostate-specific antigen, *TURP* transurethral resection of the prostate, *PVP* photoselective vaporization of the prostate

Univariate analysis detected age (*P* < 0.01, OR 1.08, 95% CI 1.04–1.12), diabetes (*P* = 0.020, OR 2.47, 95% CI 1.14–5.31), preoperative gross hematuria (*P* = 0.02, OR 0.48, 95% CI 0.27–0.87), Qmax (*P* = 0.017, OR 0.89, 95% CI 0.82–0.98), bladder compliance (*P* < 0.01, OR 3.96, 95% CI 2.17–7.24), damage of detrusor function (*P* < 0.01, OR 11.22, 95% CI 4.30–29.20), and the severity of BOO (*P* < 0.01, OR 4.08, 95% CI 2.21–7.54) as independent predictors for incomplete recovery of urination function (Table 2). After multivariate analysis, four predictors were left, age (*P* < 0.01, OR 1.07, 95% CI 1.02–1.15), bladder compliance (*P* < 0.01, OR 2.37, 95% CI 1.20–4.67), detrusor function (*P* < 0.01, OR 5.92, 95% CI 2.10–16.6), and the severity of BOO (*P* < 0.01, OR 2.21, 95% CI 1.07–4.54).

**Table 2:** Univariate and multivariate regression analyses of risk factors

Variables	Univariate analysis		Multivariate analysis	
	OR (95%CI)	*P* value	OR (95%CI)	*P* value
Age (years)	1.08 (1.04, 1.12)	<0.01	1.07 (1.02, 1.15)	<0.001
BMI (kg m^2^)	0.96 (0.84, 1.03)	0.386		
Duration (months)	1.00 (0.99, 1.01)	0.982		
Smoking	1.65 (0.91, 3.03)	0.101		
Drinking	1.26 (0.67, 2.34)	0.471		
Acute urinary retention	0.54 (0.27, 1.05)	0.073		
Medication	1.42 (0.78, 2.56)	0.245		
Glucose	1.10 (0.82, 1.48)	0.499		
Creatinine	1.00 (0.99, 1.01)	0.261		
Albumin	0.91 (0.84, 0.98)	0.026		
Urinary tract infection	1.83 (1.01, 3.32)	0.046		
Hypertension	0.83 (0.68, 2.56)	0.670		
Diabetes	2.47 (1.14, 5.31)	0.020		
PV (ml)	0.98 (0.97, 1.00)	0.061		
PVR (ml)	1.00 (0.92, 1.00)	0.304		
tPSA (ng/ml)	1.00 (0.92, 1.08)	0.996		
Preoperative gross hematuria	0.48 (0.27, 0.87)	0.016		
IPSS	1.04 (0.96, 1.14)	0.265		
Qol	1.14 (0.72, 1.83)	0.561		
Qmax	0.89 (0.82, 0.98)	0.017		
MCC (ml)	0.99 (0.98, 0.99)	<0.01		
Bladder compliance	3.96 (2.17, 7.24)	<0.01	2.37 (1.20, 4.67)	0.013
Detrusor function	11.2 (4.30, 29.2)	<0.01	5.92 (2.10, 16.6)	0.001
Bladder outlet obstruction	4.08 (2.21, 7.54)	<0.01	2.21 (1.07, 4.54)	0.030
Surgery type	1.12 (0.49, 2.59)	0.79		
OT (min)	0.99 (0.98, 1.01)	0.524		
Bladder stone	1.85 (0.68, 5.00)	0.22		
Cystic trabeculae	1.34 (0.75, 2.38)	0.31		

*BMI* Body Mass Index, *PV* Prostate Volume, *PVR* Post-void Residual, *BOO* Bladder Outlet Obstruction, *OT* operation time

Furthermore, the collinearity diagnostic analysis demonstrated that the VIFs of those risk factors were less than 4 (Supplementary Table 1), indicating that there is no strong indication of multicollinearity among variables. Thus, there were four variables included in the final multivariable prediction model as predictors.

**Fig. 2:**
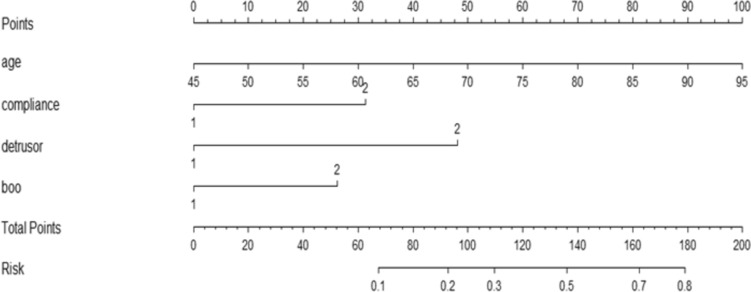
The nomogram model for prediction of efficacy in benign prostatic hyperplasia patients after transurethral surgery. For “compliance,” 1 means better compliance and 2 means lower compliance. As to “detrusor,” 1 means stronger contractility and 2 means weaker contractility. With regard to “BOO,” 1 means obvious BOO and 2 means unclear or non-existent BOO

**Fig. 3:**
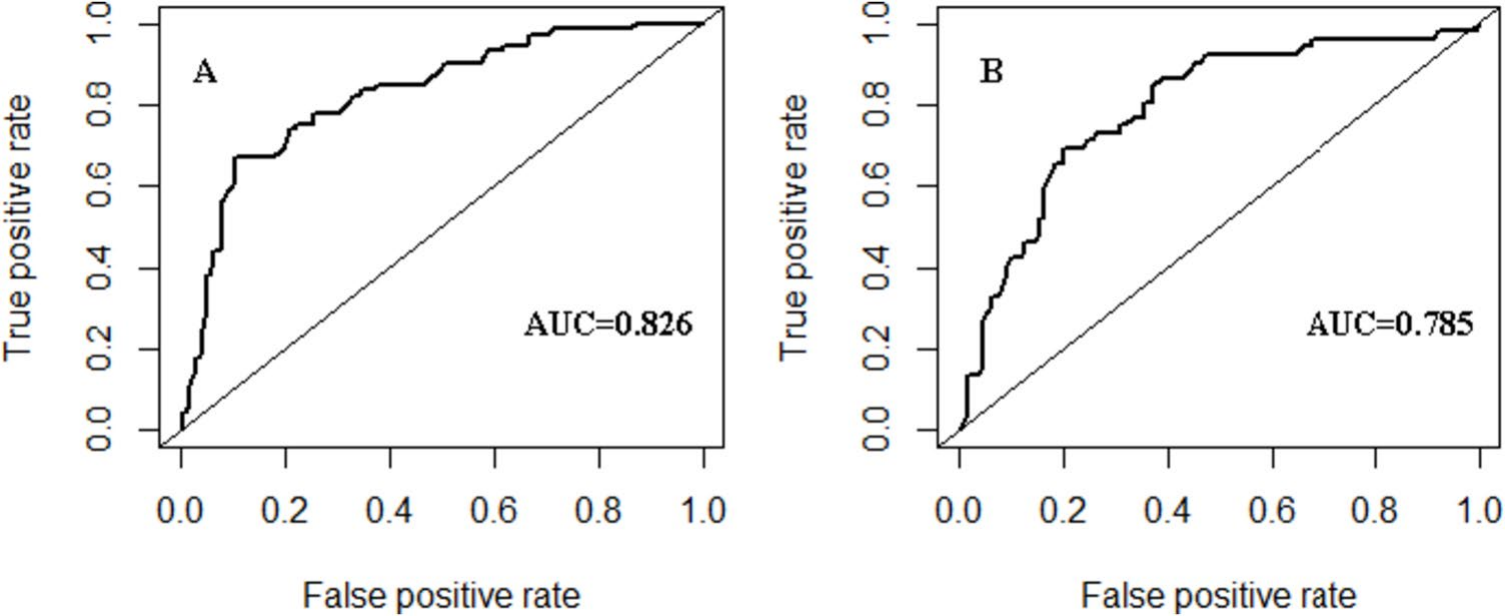
Receiver operating characteristic curves of the nomogram in the training (A) and validation (B) groups

**Fig. 4:**
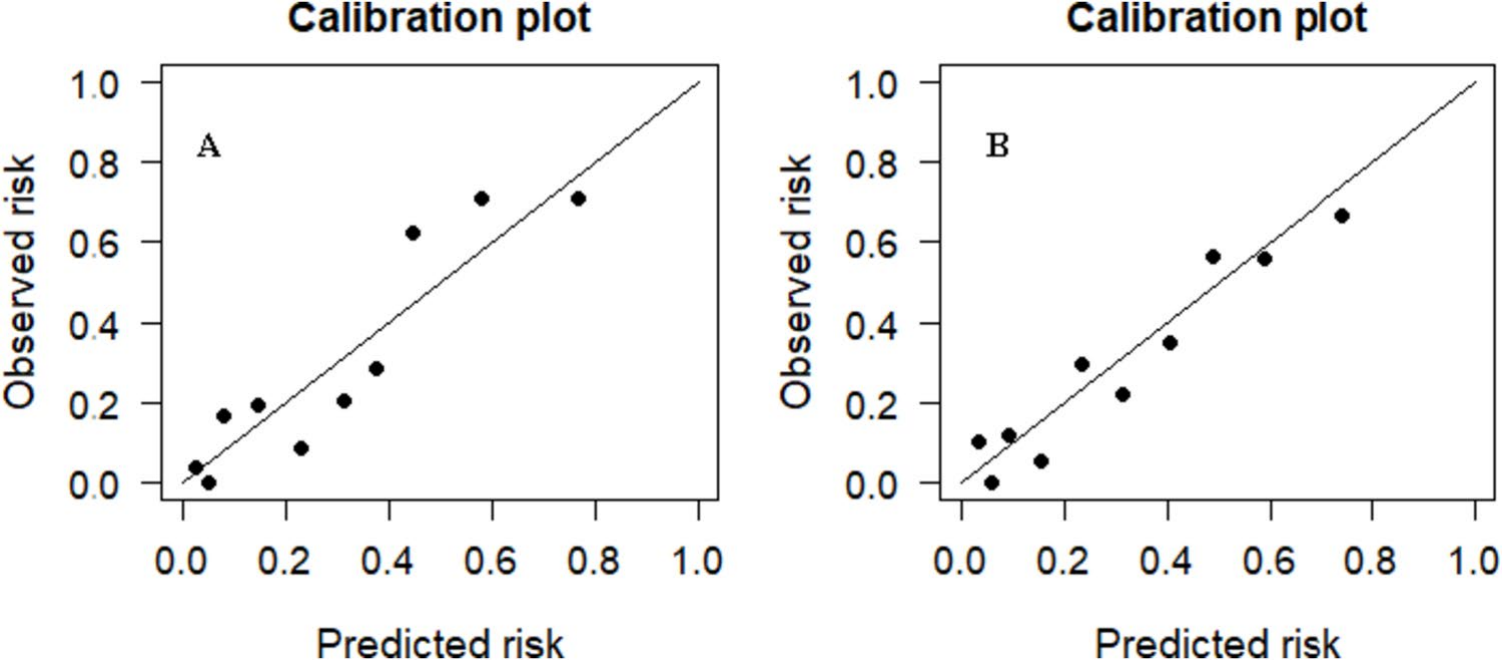
Calibration plots of the nomogram in the training (A) and validation (B) groups

The nomogram was established by assigning a graphic preliminary score to each of the 4 significant predictors with a point ranging from 0 to 100, which was then summed to generate a total point, and finally converted into an individual probability of incomplete recovery of urination function after transurethral surgery (Fig. 2). For example, a patient with age at 65 years, bladder compliance decompensation, detrusor contractility decreased, and severe bladder outlet obstruction would have a total of 71 points (40 points for age, 31 points for bladder compliance, 0 for detrusor, 0 for bladder outlet obstruction). The predicted possibility of unsatisfactory rehabilitation was less than 15% for this patient. We calculated the AUC to investigate the discrimination of the nomogram, which was 0.83 (95% CI 0.77–0.88) in the training group, which indicated a good predictive power (Fig. 3A). For each patient, higher total points indicated a higher risk of unsuccessful surgical outcome. Additionally, the risk score of 125.50 was determined as the optimal cutoff value with the maximum Youden index. The sensitivity, specificity, positive likelihood ratio, and negative likelihood ratio were 0.67 (95% CI, 0.55–0.77), 0.89 (95% CI 0.84–0.94), 6.41 (95% CI 4.03–10.22), and 0.367 (95% CI 0.264–0.51) when this cutoff was applied. Figure 4A shows the comparison of the calibration between the nomogram prediction and actual observation by using a calibration plot. The calibration plot revealed good predictive accuracy of this nomogram. An additional 181 patients were prospectively enrolled as the validation group to validate the new nomogram. The baseline characteristics of the validation group are demonstrated in Table 1. The AUC of the nomogram in the validation group was 0.785 (95% CI 0.713–0.858) (Fig. 3B), and the sensitivity, specificity, positive likelihood ratio, and negative likelihood ratio were 0.67 (95% CI 0.53–0.79), 0.80 (95% CI 0.72–0.86), 3.34 (95% CI 2.26–4.94), and 0.41 (95% CI 0.28–0.61) when the cutoff value of 125.50 was utilized. The good calibration of the nomogram was also confirmed by the validation group (Fig. 4B).

## Discussion

Early in 1993, Kuo et al. revealed that after BPH surgery, part of the patients (19%) cannot achieve a successful result and in 4.5% of patients, the symptoms even worsened [3]. They figured out 9 favorable factors to predict satisfactory surgical outcomes such as the history of acute urinary retention and urodynamically obstructive BPH. However, the history of chronic urinary retention and irritative bladder symptoms alone were identified as unfavorable factors. In recent years, with the development of urodynamics, an increasing number of studies focused on postoperative unsatisfactory urination rehabilitation in BPH patients were reported. Kim et al. revealed that preoperative detrusor underactivity correlated with poorer IPSS and Qmax improvement [4]. However, in a study reported by Myeong et al., 56 patients with BPH and detrusor underactivity were enrolled. After holmium laser enucleation (HoLEP) (24 patients) and transurethral resection of the prostate (TURP) (32 patients), both groups showed significant improvement in the total IPSS and maximal flow rate compared to preoperative characteristics. Based on the result, Myeong inferred that detrusor underactivity may not be a contraindication for HoLEP and TURP [17]. The results of these two studies seemed to be inconsistent. The main reason for such a problem may be that both studies predicted the efficacy of urination function after surgery with only one or two predictors. Nevertheless, there are many other influencing factors such as age, bladder compliance, and the history of AUR. Therefore, the prediction was not ideal. In 2015, De Nunzio et al. developed a nomogram for the diagnosis of BPH (the Young Academic Urologist nomogram, YAU nomogram), which have two prediction factors: Qmax and transitional zone volume [18]. Then YAU nomogram was applied to the prediction of clinical outcome of Italian patients treated with TURP and this nomogram achieve good results with an area under the curve of 0.77 [19]. However, only part of urodynamic factors were included in their study which means the bladder function was not fully considered. Young et al. have analyzed the data from UPSTREAM trail and pointed out that transurethral surgery was more beneficial in those with bladder outlet obstruction index > 47.6, and bladder contractility index > 123.0, which is consistent with our study [20, 21]. Our study aims to develop and validate a model for predicting the probability of postoperative urination unsatisfactory rehabilitation. And our data showed that age, bladder compliance, detrusor function, and the severity of bladder outlet obstruction were significant and independent predictors for postoperative urination unsatisfactory rehabilitation which is consistent with previous studies.

Choi et al. studied 116 men with symptomatic BPH requiring surgery, they found that patients in the persistent storage symptom-positive group were older than those in the storage symptom-negative group [22]. This finding was also confirmed by the nomogram reported by De Nunzio et al [15]. Both of them conclude that age is an independent predictor for poor TURP outcome. Previous studies have revealed that age may lead to functional changes in bladder compliance and detrusor. The multicollinearity diagnosis in our study indicated that there is no multicollinearity between age, bladder compliance, detrusor function, and BOO. Based on the results of our multicollinearity diagnosis and previous studies, we considered age as an independent predictor for poor TURP outcome.

Bladder compliance decompensation is a common function change secondary to BPH, leading to reduced capacity or increased post-void residual. Clinical studies revealed that bladder compliance significantly correlates with LUTS in the elderly population. It was shown that decreased blood flow and the changed ratio of collagens in the human bladder significantly correlate with reduced bladder compliance [23, 24]. The predictive value of bladder compliance was also validated in our study.

Detrusor underactivity is widely perceived as contributing to poor surgical treatment outcomes [4, 25]. Cho et al. pointed out that deterioration of voiding symptoms at long-term follow-up visits after HoLEP is more significant in LUTS/BPH patients with DU [26]. Currently, the possible mechanism of low detrusor contractility may be that the detrusor muscle fibers are disordered or even broken after long-term BPH which leads to a lower detrusor muscle-to-collagen ratio, and changes in collagen and muscle quantity [27]. Our data also confirmed that better detrusor contractility was associated with better surgical outcomes of TURP.

BOO is one of the most important components to assess in patients with LUTS/BPH. Currently, it is widely acknowledged that preoperative identification of BOO is the prerequisite and key point for successful postoperative surgical outcomes. Schneider et al. concluded in a meta-analysis that a significant association between urodynamic BOO and better improvements in all treatment outcome parameters such as IPSSs, QoL scores, Qmax, and PVR [28]. However, Masumori et al. reported that although the IPSS in patients without BOO deteriorated faster than in those with it, two-thirds of patients with DUA but not BOO were satisfied with their urinary condition at 12 years [29]. In a word, BOO was able to be used as a valuable predictor, but surgery was also reasonable for patients with insignificant BOO, which was consistent with our results.

## Limitation

Our study has several limitations that should be noted. First, patients who underwent TURP and PVP were included, while patients treated with HoLEP were not included, which may weaken the universality of the model. Second, although we have collected as many predictors as possible, some potential predictors associated with successful surgical outcomes were not available, such as prostatic urethral length and bladder wall thickness [30]. Third, the history of medication for LUTS/BPH was not meticulously classified, which may weaken the predictive value of this predictor. Finally, more patients with varied clinical characteristics especially larger prostate volume should be included in our training group in order to improve our prediction model.

## Conclusion

In conclusion, our nomogram composed of age, bladder compliance, detrusor contractility, and BOO may predict the efficacy of TURP and PVP. The AUC of this nomogram indicates a superior discriminative ability. The discrimination and calibration of the nomogram were confirmed in internal and external validation.

### Supplementary Information

Below is the link to the electronic supplementary material.

Supplementary file1 (DOCX 18 kb)

## Data Availability

The authors confirm that the data supporting the findings of this study are available within the article and its supplementary materials.
