# Supplementary material for: Nomogram predicting the efficacy of transurethral surgery in benign prostatic hyperplasia patients
Source: Aging Clin Exp Res. 2024 Mar 14;36(1):71. doi: 10.1007/s40520-024-02708-8 (PMC10940401; doi:10.1007/s40520-024-02708-8)
Supplement: Supplementary file 1 — Supplementary file1 (DOCX 18 kb) [file 40520_2024_2708_MOESM1_ESM.docx]

Supplementary table 1 The tolerance and variance inflation factor (VIF) for the variables in the nomogram

|  | Training Group | |  | Validation Group | |
| --- | --- | --- | --- | --- | --- |
| Variables | tolerance | VIF |  | tolerance | VIF |
| Age | 0.935 | 1.696 |  | 0.937 | 1.608 |
| Compliance | 0.901 | 1.110 |  | 0.912 | 1.097 |
| Detrusor | 0.706 | 1.417 |  | 0.857 | 1.166 |
| BOO | 0.751 | 1.332 |  | 0.842 | 1.188 |

VIF: variance inflation factor ; BOO: bladder outlet obstruction
